# Association of a Novel Nonsense Mutation in KIAA1279 with Goldberg-Shprintzen Syndrome

**Published:** 2017

**Authors:** Shadab SALEHPOUR, Feyzollah HASHEMI-GORJI, Ziba SOLTANI, Soudeh GHAFOURI-FARD, Mohammad MIRYOUNESI

**Affiliations:** 1 Department of Pediatrics, Mofid Children Hospital, Faculty of Medicine, Shahid Beheshti University of Medical Sciences, Tehran, Iran; 2 Genomic Research Center, Shahid Beheshti University of Medical Sciences, Tehran, Iran; 3 Department of Medical Genetics, Shahid Beheshti University of Medical sciences, Tehran, Iran

**Keywords:** Goldberg-Shprintzen syndrome, KIAA1279, Mutation

## Abstract

Goldberg-Shprintzen syndrome (OMIM 609460) (GOSHS) is an autosomal recessive multiple congenital anomaly syndrome distinguished by intellectual disability, microcephaly, and dysmorphic facial characteristics. Most affected individuals also have Hirschsprung disease and/or gyral abnormalities of the brain. This syndrome has been associated with KIAA1279 gene mutations at 10q22.1. Here we report a 16 yr old male patient referred to Center for Comprehensive Genetic Services, Tehran, Iran in 2015 with cardinal features of GOSHS in addition to refractory seizures. Whole exome sequencing in the patient revealed a novel nonsense (stop gain) homozygous mutation in KIAA1279 gene (KIAA1279: NM_015634:exon6:c.C976T:p.Q326X). Considering the wide range of phenotypic variations in GOSHS, relying on phenotypic characteristics for discrimination of GOSH from similar syndromes may lead to misdiagnosis. Consequently, molecular diagnostic tools would help in accurate diagnosis of such overlapping phenotypes.

## Introduction

Goldberg-Shprintzen syndrome (OMIM 609460) (GOSHS) is a multiple congenital anomaly syndrome inherited in an autosomal recessive manner and first described by Goldberg and Shprintzen in 1981 (1) and referred as Goldberg-Shprintzen syndrome (2). It is distinguished by intellectual disability, microcephaly, and dysmorphic facial characteristics. The majority of affected persons suffer from Hirschsprung disease and/or gyral defects in the brain, as the results of shortcomings in migration of neurons during embryogenesis. Additionally, megalocornea or urogenital anomalies may be detected in patients. The phonotypic features resembles Mowat-Wilson syndrome (MOWS; 235730) but these two disorders are genetically distinctive (3). GOSHS has been associated with KIAA1279 gene mutations at 10q22.1 (4). KIAA1279 encodes KIF binding protein (KBP). KBP has two tetratricopeptide repeats (TPRs) that are structuralmotifs involved in protein–protein interactions (5). KBP has been shown to be universally and pervasively expressed in early development while overexpressed in the central and enteric nervous systems in later stages (6). It interacts with the actin andtubulin cytoskeleton. In addition, its expression directly influences neurite growth in a neuron-like cell line in accordance with the central and enteric neuronal developmental deficiencies observed in GOSHS patients (3). 

To date, seven different KIAA1279 mutations has been described in GOSHS patients all of which being nonsense mutations (Table 1). 

## Case Report

**Fig 1:**
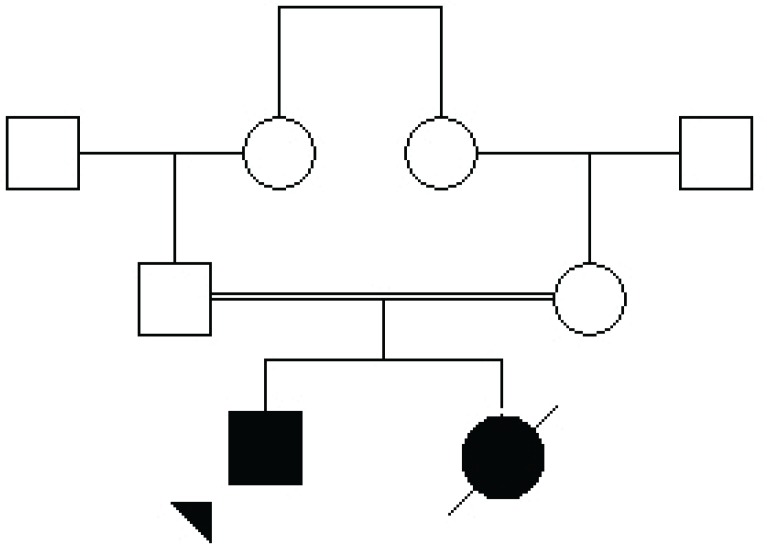
Family pedigree showing the consanguinity of patients’ parents

The patient is a 16 yr old boy referred to Center for Comprehensive Genetic Services, Tehran, Iran in 2015. He was the first child of healthy first-cousin Iranian parents whose second child had congenital microcephaly and hypotonia and died at the age of 12 months without any specific diagnosis (Figure 1). The proband was delivered by a normal vaginal delivery at 40 weeks of gestation after an unremarkable pregnancy. His birth occipito-frontal head circumference (OFC) was 30 cm which was 3 standard deviations (SD) lower than the age, sex and ethnicity matched population average. Afterwards, hypotonia, delayed developmental milestones as well as seizures and mental retardation were reported. 

A history of bilateral cryptorchidism, inguinal hernia and Hirschsprung disease was reported which were surgically corrected. At the age of 16, his OFC was 52.5 cm which was in normal range. In physical exam he had gait disturbance, facial dysmorphism, speech defect, excessive drooling, spasticity and dry eyes. Cytogenetic analysis of the patient showed numerically normal male karyotype. The brain magnetic resonance imaging (MRI) of the patient showed brain atrophy as well as corpus callosum hypoplasia. 

Informed consents were obtained from parents before participation in the study in accordance with the protocol approved by local institutional Ethics Committee. 

**Fig 2:**
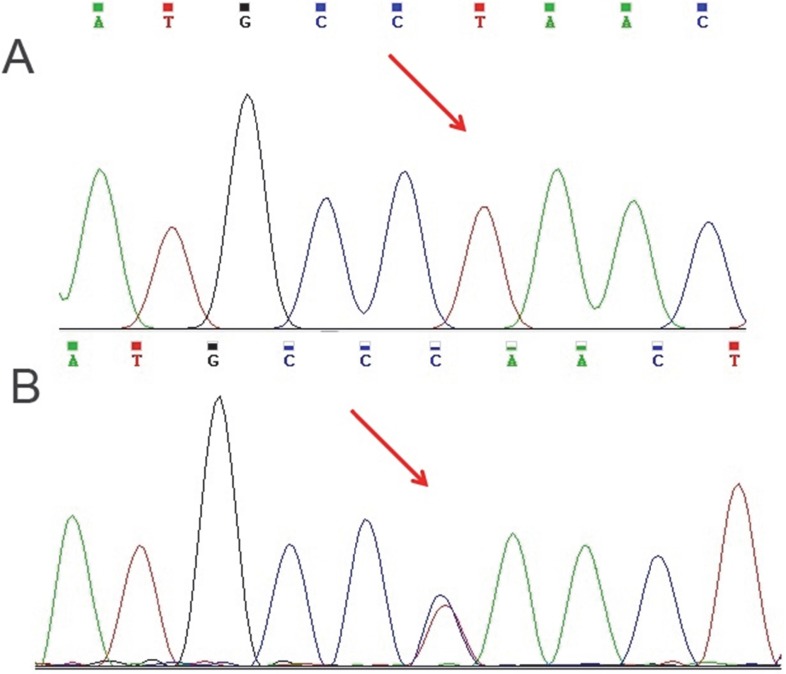
Nucleotide change in KIAA1279 gene in patient (A) and his parents (B

Blood samples were collected from patient and his parents in EDTA tubes. DNA was isolated using the standard salting out method. Whole exome sequencing was performed using Illumina’s Genome Analyzer for the patient with focus on 2752 OMIM disease genes (BGI-Clinical Laboratories, Shenzhen, China). The results were verified by Sanger sequencing using the ABI Prism3130 Genetic Analyzer (Applied Biosystems, Foster City, CA, USA). A novel nonsense (stop gain) homozygous mutation was detected in KIAA1279 gene (KIAA1279: NM_015634:exon6:c.C976T:p.Q326X) correlated with the observed phenotype. This variant has not been reported in generalist polymorphism databases (ExaC or exome variant server (EVS)), dbSNP and 1000 genome project. Combined Annotation Dependent Depletion (CADD) tool which is a tool for scoring the deleteriousness of single nucleotide variants as well as insertion/deletions variants in the human genome indicated that this variant would be deleterious with a score of 17.21. Targeted sequencing on the parents showed the expected segregation pattern (Figure 2).

## Discussion

GOSHS is a condition first defined in patients with syndromic Hirschsprung disease and attributed to neuralcrest developmental defects. Further reports of bilateral polymicrogyria in patients with KIAA1279 mutations implied that the encoded protein participates in cerebral cortex development as well (3). 

**Table 1:** Detected Mutations in KIAA1279 Gene

Mutation	Exon number	Ethnicity	Clinical features other than classical features	Reference
c.G250T (E84X)	Exon 1	British Pakistani	Coloboma	(4)
c.C268T (R90X)	Exon 1	Moroccan	No Hirschsprung disease in some cases, corneal hypoesthesia, strabismus, syndactyly, conductive hearing loss, proximal muscle weakness, megalocornea	(3, 4)
EX2-3DEL (N143fsX1)	Exons 2 and 3	Pakistani	Fetal polymicrogyria, microcephaly, hypoplastic corpus callosum, No Hirschsprung disease, No cardinal signs of GOSHS	(7)
c.C599A (S200X)	Exon 3	French, Moroccan	Ptosis, hyperopia, aortic valve incompetence, cryptorchidism, high palate, oligodontia, scoliosis, vesicoureteral reflux, multicystic renal dysplasia, femoral neck anteversion	(3)
c.604_605delAG (R202IfsX2)	Exon 3	Iraqi	Ventricular septal defect	(3)
c.1116_1117insA (Y466X)	Exon 7	Not mentioned	Foot anomalies including camptodactyly and clinodactyly	(8)
c.C976T(Q326X)	Exon 6	Iranian	Inguinal hernia, criptorchidism, seizure	The present study

In this report, we have demonstrated that a novel nonsense mutation in KIAA1279 gene is associated with GOSHS in a consanguineous family. Although Hirschsprung disease is not a consistent manifestation of this syndrome, the patient reported in the present study suffered from it. However, microcephaly as a frequent manifestation of this syndrome has been reported in the patient in infancy but current physical examination showed normal OFC in contrast with a previous report (7). As different mutations in the mentioned gene have been associated with distinct phenotypes, genotypephenotype correlation can be established for diverse mutations leading to this syndrome. Previously reported mutations in this gene have been stop gain mutations leading to nonsense-mediated mRNA decay and loss of KBP function. The ubiquitous expression of KBP predicts that such mutations result in a wide range of phenotypic manifestation in various organs. 

The observed phenotypic features in this patient including mental disturbances, seizures, and gastrointestinal as well as urogenital abnormalities are in accordance with this assumption. Another prominent feature of the patient has been his refractory seizure from his infancy, which had not been reported in GOSHS patients previously assumed as a distinguishing feature between GOSHS and MOWS (7).

**In conclusion,** the present report indicates that relying on phenotypic characteristics for discrimination of these two somehow similar syndromes may lead to misdiagnosis. Consequently, molecular diagnostic tools would help in accurate diagnosis of such overlapping phenotypes.
